# Advantage of vacuum assisted closure on healing of wound associated with omentoplasty after abdominoperineal excision: a case report

**DOI:** 10.1186/1477-7819-6-136

**Published:** 2008-12-23

**Authors:** Silvia Cresti, Mehdi Ouaïssi, Igor Sielezneff, Jean-Baptiste Chaix, Nicolas Pirro, Bruno Berthet, Bernard Consentino, Bernard Sastre

**Affiliations:** 1 Service de Chirurgie Digestive et Oncologique, Pôle d'Oncologie et de Spécialités Médicales et Chirurgicales, Hôpital De la Timone, Marseille, France

## Abstract

**Background:**

Primary closure of the perineum with drainage after abdominoperineal excision of the rectum for carcinoma, is widely accepted. However hematoma, perineal abscess and re-operation are significantly more frequent after primary closure than after packing of the perineal cavity. Those complications are frequently related to the patients' clinical antecedent (i.e radiotherapy, diabetes, smoking).

**Case presentation:**

In the present report, vacuum assisted drainage was used after abdominoperineal excision for carcinoma in the very first step due to intraoperative gross septic contamination during tumor resection. The first case: A 57-years old man with a 30-years history of peri-anal Crohn's disease, the adenocarcinoma of the lowest part of the rectum and Crohn colitis with multiple area of severe dysplasia required panproctocolectomy with a perineal resection. The VAC system was used during 12 days (changed every 3 days). We observed complete healing 18 days after surgery. The second case: A 51-year-old man, with AIDS. An abdominoperineal resection was performed for recurrence epidermoid anal cancer. The patient was discharged at day 25 and complete healing was achieved 30 days later after surgery.

**Conclusion:**

The satisfactory results showed in the present report appear to be favored by association of omentoplasty and VAC system. Those findings led us to favor VAC system in the case of pelvic exenteration associated with high risk of infection.

## Background

Primary closure of the perineum with drainage after abdominoperineal resection (APR) of the rectum for carcinoma, is widely accepted [1]. Meticulous hemostasis and avoidance of intra-operative gross septic contamination are mandatory. However hematoma, perineal abscess and reoperation are significantly more frequent after primary closure than after packing of the perineal cavity[1]. Those complications are frequently related to the patients' clinical antecedent (i.e. radiotherapy, diabetes, smoking) [2-4]. Thus, failure of perineal wound healing after adenocarcinoma of the lower rectum is a major problem in colorectal surgery. It prolongs hospitalization and may delay or even preclude adjuvant radiochemotherapy with a direct impact on local recurrence and long-term survival [5]. In our experience as Debroux study's, we usually use transposition of great omentum in APR with excellent primary perineal wound healing [6]. Vacuum Assisted-Closure (VAC^®^: KCI Kinetic Concept Inc, San Antonio, Texas) device decreases the time of wound healing, thus increasing the deposition of granulation tissue [7]. Thus, we decided to replace dressing by VAC^®^. We report, for the first time, safety of this management in order to improve and reduce the long stay of hospitalization as well as the development of chronic perineal sinus. This first preliminary observation of wound dehiscence management after APR using transposition of greater omentum and VAC to be extended in a large scale requires a prospective study. Moreover, such study may allow to investigate the possible benefit of the method we have described in the present report to increased angiogenesis.

## Case presentation

### Case 1

**Figure 1:**
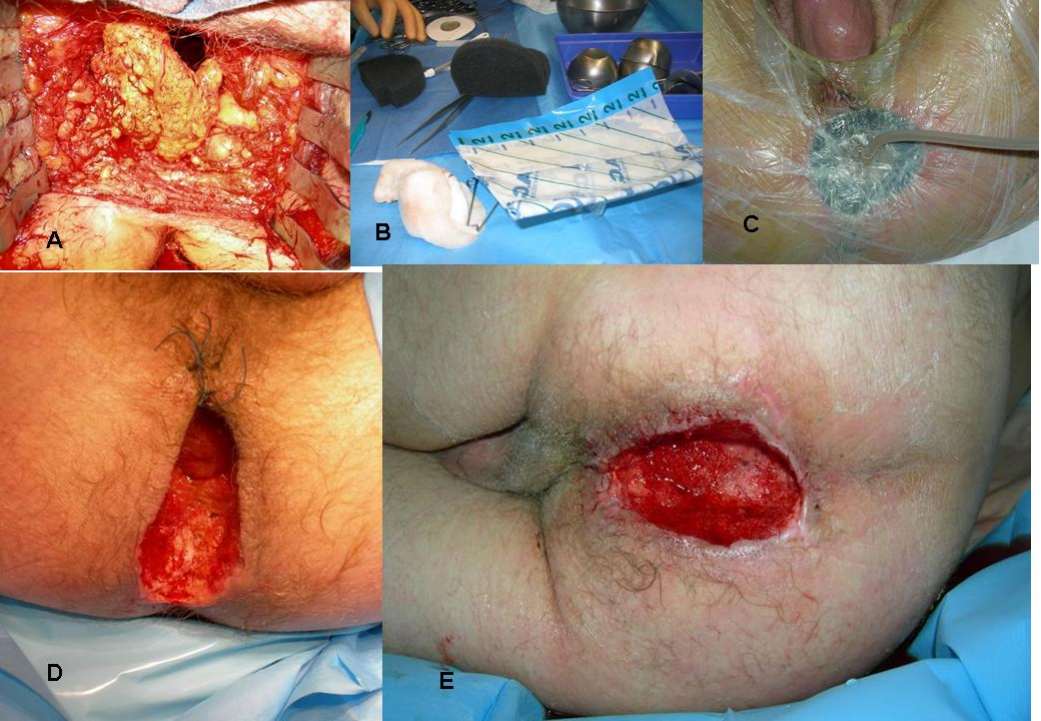
**A: Pedicled omentum is sutured to the subcutaneous fatty tissue with slowly absorbable interrupted sutures**. **B**: Vacuum-assisted closure system **C**: Suction apparatus **D**: Perineal wound after 3 days of VAC^® ^treatment at 100 mmHg. Note the contracted wound with healthy granulation tissue. **F**: Perineal wound after 10 days of VAC^® ^treatment at 100 mmHg. Note the contracted wound with healthy granulation tissue.

A 57-years old man with a 30-years history of perianal Crohn's disease, reported, after a long lasting treatment of his perianal disease recurrence, changes in symptoms such as bleeding per rectum with tenesmous. Instrumental examination (coloscopy and total body computed tomography) found an anal verge tumor of 1,5 cm size and a severe chronic colitis (Crohn's colitis). Staging of magnetic resonance imaging (MRI) was T2N0 and confirmed by ultrasonographic endoscopy. A computed tomography scan of the chest and abdomen was normal. Adenocarcinoma of the lowest part of the rectum and multiple area with severe dysplasia were assessed by pathological examination. For the first case, panproctocolectomy combined with perineal resection was indicated for the development of malignancy and synchronous multiple area of dysplasia in the background of chronic severe colitis. The rectum was removed according to TME (total mesorectum excision) principles [8]. The omentum was divided and delivered to the pelvic cavity and the pelvic peritoneum was closed through the abdomen (Figure 1). Septic contamination occurred by leakage of fecal material from the anus during the perineal dissection. Moreover, there was a perianal chronic sepsis due to Crohn'disease.

The perineal cavity was not primarily closed, but packed with four roll gauze in order to ensure the perinaeal hemostasis prior to put VAC^® ^in place. The pack was removed one day later and the VAC^® ^was settled in place (the wound measured 10 cm × 10 cm) under general anesthesia and set at 100 mmHg depression (Type of foam was **V.A.C.^® ^**GranuFoam^® ^Medium Dressing Kit). Suction was chosen in function of perineal pain. The pressure of suction was applied in the absence of perineal pain. The VAC^® ^was changed every 72 hours by nurses under local anesthesia. Twelve days later, a significant reduction of the wound size (4 cm × 6 cm) was evident and the VAC^® ^procedure was stopped. Amount of fluid was 300 cc every day during five days and decreased to 200 cc during 3 days and 30 cc during the last two days. The patient had a remarkable recovery and was discharged at day 13th after surgery. The second treatment was made by the nurse and consisted of sterile alginate dressings-Algosteril (Brothier Laboratories) every day during five days. A complete healing was achieved within 18 days after surgery

Pathologic examination showed a rectal adenocarcinoma, staged pT3 N1 M0 R0 with complete mesorectal excision and 2 mm of circumferential resection margin. These results led to the onset of an adjuvant systemic therapy including radiotherapy (45 Gy) and chemotherapy (Leucovorin-5 FU). Radiotherapy was conducted due to the T3 local invasion and invaded nodes as well as septic contamination.

### Case 2

A 51-year-old man, with AIDS, previously treated for Hodgkin's disease, developed a local recurrence six years after the treatment of an anal epidermoid cancer, initially managed by chemoradiation therapy (60 Gy and 5-fluorouracil and mitomycin C) one year before. He was classified stage IV according to WHO (World Health Organization clinical staging), and staging C following the Center for disease control (CDC) classification. The CD4 cell count was 190 cells/μl. Patient was treated by stavudine (anti-retroviral drug) more than 5 years. Recurrence of anal epidermoid cancer was staged in MRI T4N+. There was no distant metastase in thoraco-abdominal computed tomography. An abdominoperineal resection was conducted. The great omentum was pediculized on the left gastroepiplooic artery and tightly sewn to the subcutaneous fatty tissue of the perianeal skin. The perineum was not closed primarily, but packed with three roll gauzes. Septic contamination occurred by leakage of fecal material from the anus during the perineal dissection.

**Figure 2:**
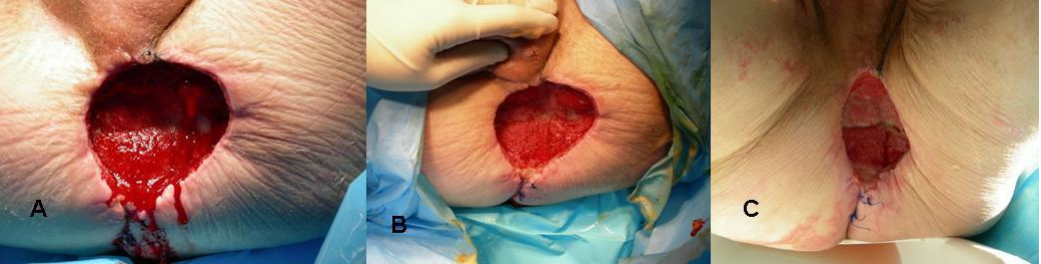
**A: Perineal wound after 3 days of VAC^® ^continuous treatment at 125 mmHg**. **B**: Perineal wound after 8 days of VAC^® ^continuous treatment at 125 mmHg. Note the contracted wound with healthy granulation tissue **C**: Perineal wound after 12 days of VAC^® ^continuous treatment at 125 mmHg. Note the contracted wound with healthy granulation tissue.

One day after surgery, the pack was removed and the VAC^® ^system was left in place under general anesthesia (the wound measured 20 cm × 17 cm) and set at 125 mmHg suction (figure 2). As in the case described above, suction was chosen in function of perineal pain. The pressure of suction was applied in the absence of perineal pain. Type of foam was **V.A.C.^® ^**GranuFoam^® ^Medium Dressing Kit. The VAC^® ^was changed every 48 hours by nurses under local anesthesia. The amount of fluid was 500 cc every day during fifteen days and decreased to 400 cc during 5 days and 100 cc during the last three days. We have experienced the tight of dressing wound to be difficult when the amount of count fluid reached values near 500 cc. Therefore, we have shortened the VAC change period to 48 h.

Twenty days later, a significant reduction of the wound (12 cm × 9 cm) was observed. The stage of the tumor was pT3N0M0 R0. A survey without adjuvant chemotherapy was applied. The patient was discharged at day 25 and complete healing was achieved 30 days later. The second treatment was made by the nurse and consisted of sterile alginate dressings-Algosteril (Brothier Laboratories) every day during five days.

## Discussion

Failure of perineal wound healing after adenocarcinoma of the lower rectum is a major problem in colorectal surgery. It prolongs hospitalization and may delay or even preclude adjuvant radiochemotherapy with a direct impact on local recurrence and long-term survival [5]. Various surgical options have been reported to manage the perineal wound after abdomino-perineal rectal resection: 1-closure with drainage; 2-reconstruction with plastic surgery; 3-packing [2-4].

In Delalande'report, patients with sepsis contamination or unsatisfactory hemostasis were enrolled in randomized study[1]. Primary closure was associated with a significantly higher rate of healed perineums at one month (30 percent *vs. 0 *percent; P = 0.01) and a shorter delay to complete cicatrization (median, 47 *vs. *69 days) (P < 0.01). Conversely, hematoma, perineal abscess, and re-operations were significantly more frequent (P < 0.01) in the primary closure group[1]. Delande's study was used as a reference in packing since it represents the unique randomized study including sufficient number of patients and having conducted packing and primary wound in patients with sepsis contamination or unsatisfactory haemostasis [1].

Moreover, the reconstruction by well vascularized tissue in large pelvic exenteration had the same risk of disunion or wound abscess of expert Team and are represented by patients with pelvic exenteration and septic contamination[9]. According to Butler's recent retrospective study, VRAM flap reconstruction of irradiated APR defects reduces major perineal wound complications without increasing early abdominal wall complications[10]. To our knowledge, there is no randomized study which compared the reconstruction of well vascularized bulky tissue between packing or wound closure in patients with sepsis contamination or unsatisfactory hemostasis. Thus, in specific situation (i.e. radiotherapy, sepsis contamination or pelvic exenteration) we preferred used the physiologic properties of the omentum and not to conduct primary closure.

As in Debroux's study we usually use transposition of great omentum in APR with excellent primary perineal wound healing[6]. According to Christian study's patients with anal cancer and inflammatory bowel disease were at higher risk for perineal wound complications than those with rectal cancer. Vacuum assisted closure may be successfully used after complex perineal wound (such as Fournier's gangrene) or after persistent perineal sinus[11,7]. The Vacuum-assisted closure device decreases the time of wound healing, thus increasing the deposition of granulation tissue [7]. In the present study, we reported for the first time the dressing replacement by VAC which might be an interesting approach leading to decrease hospitalization duration and reduction of chronic perineal sinus development.

Several factors led us to use VAC therapy in the present reported cases:

- A safe and dry dressing is difficult to achieve after packing (Mickulicz) [7];

- The presacral space left after rectal excision enables accumulation of blood and effusion enhancing therefore the potential risk of wound infection [7];

- Patients' antecedent (Crohn's disease) or immunodeficiency (HIV) potentially increased the wound infection risk that might interfere with perineal closure.

- Avoidance of chronic sinus due to wide abdomino-perineal resection [12].

Moreover, during VAC therapy a significant change in bacterial local flora (decrease number of non fermentative bacteria) and diminution of bacteria count were observed [13,14]. These findings appear to favor healing and might shorten the length of the hospitalization stay.

In the De Broux's study healing was not defined[6]. A number of studies have reported the rate of abscess, disunion, or event which delayed healing. However, the cicatrization evolution was not defined. According to De Broux's study[6], the length of hospitalization stay was 20 ± 9 days, and this value is comparable to that observed in the present report (18, 25 days). Moreover, due to the fact that it is a new management of abdomino perineal resection study we decided to have a complete healing for the hospital discharge. The second treatment was made by the nurse and consisted of sterile alginate dressings-Algosteril (Brothier Laboratories) every day during five days after discharge for the two patients. In view of the data, the management of the large tissue defects in pelvic regions by means of VAC as a temporary coverage positively supports wound conditioning, reduces infectious complications, and facilitates a definitive wound closure [14]. The efficacy of VAC^® ^in pelvic resection in cirrhotic patient [15] was confirmed by Stawicky et al. Thus, Vacuum-based therapy appears to be safe, effective, and convenient to the patient and nursing staff, and allows for less frequent dressing changes and better quantification of fluid loss from the wound [15].

## Conclusion

Although our preliminary observations are related to two patients, it is likely that the association of omentoplasty and VAC system is the key factor leading to the satisfactory results reported in the present study. These findings led us to favor VAC system in case of pelvic exenteration associated with high risk of infection.

## Consent

Written informed consent was obtained from the patients for publication of this case report and accompanying images. A copy of the written consent is available for review by the Editor-in-Chief of this journal.

## Competing interests

The authors declare that they have no competing interests.

## Authors' contributions

SC was involved in study concept and design, acquisition of data, analysis and interpretation of data, and drafting of manuscript. MO was involved in study concept and design, acquisition of data, analysis and interpretation of data, and critical revision of manuscript, study supervision. IS was involved in study concept and design, analysis and interpretation of data, and critical revision of manuscript. JC was involved in acquisition of data. NP was involved in the drafting of manuscript. BB was involved in critical revision of manuscript.

BC was involved in critical revision of manuscript. BS was involved in study concept and design, drafting of manuscript and its critical revision for important intellectual content with over all study supervision.

## References

[B1] Delalande JP, Hay JM, Fingerhut A, Kohlmann G, Paquet JC (1994). Perineal wound management after abdominoperineal rectal excision for carcinoma with unsatisfactory hemostasis or gross septic contamination: primary closure vs. packing. A multicenter, controlled trial. French Association for Surgical Research. Dis Colon Rectum.

[B2] Bullard KM, Trudel JL, Baxter NN, Rothenberger DA (2005). Primary perineal wound closure after preoperative radiotherapy and abdominoperineal resection has a high incidence of wound failure. Dis Colon Rectum.

[B3] Chessin DB, Hartley J, Cohen AM, Mazumdar M, Cordeiro P, Disa J, Mehrara B, Minsky BD, Paty P, Weiser M, Wong WD, Guillem JG (2005). Rectus flap reconstruction decreases perineal wound complications after pelvic chemoradiation and surgery: a cohort study. Ann Surg Oncol.

[B4] Christian CK, Kwaan MR, Betensky RA, Breen EM, Zinner MJ, Bleday R (2005). Risk factors for perineal wound complications following abdominoperineal resection. Dis Colon Rectum.

[B5] Rothenberger DA, Wong WD (1992). Abdominoperineal resection for adenocarcinoma of the low rectum. World J Surg.

[B6] De Broux E, Parc Y, Rondelli F, Dehni N, Tiret E, Parc R (2005). Sutured perineal omentoplasty after abdominoperineal resection for adenocarcinoma of the lower rectum. Dis Colon Rectum.

[B7] Schaffzin DM, Douglas JM, Stahl TJ, Smith LE (2004). Vacuum-assisted closure of complex perineal wounds. Dis Colon Rectum.

[B8] Heald RJ (1995). Rectal cancer: the surgical options. Eur J Cancer.

[B9] Khoo AK, Skibber JM, Nabawi AS, Gurlek A, Youssef AA, Wang B, Robb GL, Miller MJ (2001). Indications for immediate tissue transfer for soft tissue reconstruction in visceral pelvic surgery. Surgery.

[B10] Butler CE, Gundeslioglu AO, Rodriguez-Bigas MA (2008). Outcomes of immediate vertical rectus abdominis myocutaneous flap reconstruction for irradiated abdominoperineal resection defects. J Am Coll Surg.

[B11] Yousaf M, Witherow A, Gardiner KR, Gilliland R (2004). Use of vacuum-assisted closure for healing of a persistent perineal sinus following panproctocolectomy: report of a case. Dis Colon Rectum.

[B12] Pemberton JH (2003). How to treat the persistent perineal sinus after rectal excision. Colorectal Dis.

[B13] Moues CM, Vos MC, Bemd GJ van den, Stijnen T, Hovius SE (2004). Bacterial load in relation to vacuum-assisted closure wound therapy: a prospective randomized trial. Wound Repair Regen.

[B14] Mullner T, Mrkonjic L, Kwasny O, Vecsei V (1997). The use of negative pressure to promote the healing of tissue defects: a clinical trial using the vacuum sealing technique. Br J Plast Surg.

[B15] Stawicki SP, Schwarz NS, Schrag SP, Lukaszczyk JJ, Schadt ME, Dippolito A (2007). Application of vacuum-assisted therapy in postoperative ascitic fluid leaks: an integral part of multimodality wound management in cirrhotic patients. J Burns Wounds.

